# BECC-engineered live-attenuated *Shigella* vaccine candidates display reduced endotoxicity with robust immunogenicity in mice

**DOI:** 10.21203/rs.3.rs-4448907/v1

**Published:** 2024-06-11

**Authors:** Matthew E Sherman, Jane Michalski, Sayan Das, Hyojik Yang, Lakshmi Chandrasekaran, Timothy R O’Meara, David J Dowling, Ofer Levy, Shoshana Barnoy, Malabi Venkatesan, Robert K Ernst

**Affiliations:** 1 University of Maryland-Baltimore, Department of Microbial Pathogenesis, Baltimore, MD 21201 USA; 2 University of Maryland School of Medicine, Institute for Genome Sciences, Baltimore, MD 21201 USA; 3 Walter Reed Army Institute of Research, Department of Diarrheal Disease Research, Bacterial Disease Branch, Silver Spring, MD 20910 USA; 4 *Precision Vaccines Program*, Boston Children’s Hospital, Boston, MA 02115 USA; 5 Department of Pediatrics, Harvard Medical School, Boston, MA 02115 USA; 6 Broad Institute of MIT & Harvard, Cambridge, MA 02142 USA

**Keywords:** LPS, O-antigen, lipid A, BECC, endotoxicity, live-attenuated vaccine, TLR4, *Shigella*

## Abstract

*Shigella spp*. infection contributes significantly to the global disease burden, primarily affecting young children in developing countries. Currently, there are no FDA-approved vaccines against *Shigella,* and the prevalence of antibiotic resistance is increasing, making therapeutic options limited. Live-attenuated vaccine strains WRSs2 (*S. sonnei*) and WRSf2G12 (*S. flexneri* 2a) are highly immunogenic, making them promising vaccine candidates, but possess an inflammatory lipid A structure on their lipopolysaccharide (LPS; also known as endotoxin). Here, we utilized bacterial enzymatic combinatorial chemistry (BECC) to ectopically express lipid A modifying enzymes in WRSs2 and WRSf2G12, as well as their respective wild-type strains, generating targeted lipid A modifications across the *Shigella* backgrounds. Dephosphorylation of lipid A, rather than deacylation, reduced LPS-induced TLR4 signaling *in vitro* and dampened endotoxic effects *in vivo*. These BECC-modified vaccine strains retained the phenotypic traits of their parental strains, such as invasion of epithelial cells and immunogenicity in mice without adverse endotoxicity. Overall, our observations suggest that BECC-engineered live attenuated vaccines are a promising approach to safe and effective *Shigella* vaccines.

## Introduction

*Shigella* are Gram-negative bacteria known to cause diarrheal disease in humans through ingestion of contaminated food and water^1–4^. Of the four pathogenic *Shigella* species (*S. sonnei*, *S. flexneri*, *S. boydii*, and *S. dysenteriae*), *S. sonnei* (*Ss*) and *S. flexneri* (*Sf*) cause the majority of disease in industrialized and developing countries, respectively^5^. Shigellosis (*Shigella*-induced diarrhea) affects all age groups but particularly plagues young children as uncontrolled inflammation and severe dehydration can lead to growth abnormalities, seizure, and even death^2,6–9^. The clinical severity^10,11^ and emergence of antibiotic resistance^12,13^ have prompted the development of multiple *Shigella* vaccine candidates that are currently in preclinical and clinical phases^4,14^. However, to date, there is no FDA-licensed *Shigella* vaccine.

Protection against shigellosis is serotype-specific^4,5,14,15^, implicating the O-antigen component of lipopolysaccharide (LPS) as being the critical antigen for vaccine development. Vaccine strategies to prevent shigellosis have included glycoconjugate vaccines where the O-antigen is conjugated to carrier adjuvants^4,5,14–16^ or a complex comprising LPS and the invasion plasmid antigens (Ipa) called Invaplex^17–19^. Another longstanding strategy has been the development of live-attenuated *Shigella* strains where the O-antigen remains in its native context on the outer membrane^4,5,14,15,20^. Current live-attenuated vaccine candidates focus primarily on *S. sonnei* and *S. flexneri* serotype 2a, which are epidemiologically important *Shigella* strains^21^ and whose O-antigen structures are well characterized^15^.

A key advantage of live-attenuated *Shigella* vaccines, which generally contain genetic mutations or deletions in virulence-associated genes^5,14^, is that they mimic a natural *Shigella* infection and, therefore, generate an immune response that protects against future infection. While these vaccines induce protective immune responses against virulent challenge in volunteer studies^22^, significant issues including adverse reactogenicity have slowed the progress toward a universally accepted safe and effective *Shigella* vaccine^4,5,14^. For this study, we used the live-attenuated vaccine candidates WRSs2 (*S. sonnei* strain) and WRSf2G12 (*S. flexneri* 2a strain), developed by Walter Reed Army Institute of Research (WRAIR), which are principally attenuated by deletion of *virG* (also known as *icsA*) thereby prohibiting intercellular spread but also contain deletions in genes encoding enterotoxins^23–31^. WRSs2 and WRSf2G12 are second-generation vaccines whose first-generation counterparts (WRSS1 and SC602, respectively) were highly immunogenic in adults and children during clinical trials, but substantial reactogenicity was observed at the moderate to high doses required to confer protective immunity^22,32–38^. To improve the safety of WRSs2 and WRSf2G12, we chose to target the highly immunostimulatory LPS molecule present on the bacterial membrane, which is thought to be a major contributor to these adverse effects.

LPS is a glycolipid present on the outer membrane of Gram-negative bacteria and is composed of three regions: the O-antigen, core oligosaccharide, and lipid A membrane anchor^39,40^. Innate immune cells can recognize the lipid A region of LPS and initiate cytokine production to alert the immune system to the presence of a bacterial invader^41^. This occurs through a series of accessory proteins that guide the binding of lipid A to the TLR4/MD-2 receptor on the surface of innate immune cells to drive downstream signaling, such as the NF-*κ*B pathway, and induce pro-inflammatory cytokine production^42,43^. The TLR4/MD-2 response is primarily driven by the structural features of lipid A, which vary across Gram-negative bacteria^41,42,44,45^. *Shigella* synthesizes a prototypical lipid A structure comprised of six acyl chains (hexa-acylated) and two terminal phosphates (bis-phosphorylated)^41^. This structure is a potent stimulator of TLR4/MD-2^46^, and the ensuing pro-inflammatory cytokine production likely contributes to the febrile symptoms observed upon oral ingestion of *Shigella* for vaccination. Thus, the immunostimulatory lipid A moiety was the primary target for detoxification.

In this study, we employed bacterial enzymatic combinatorial chemistry (BECC)^47^ to detoxify WRSs2 and WRSf2G12, as well as their respective wild-type strains. Lipid A modifying enzymes LpxE (1-position phosphatase from *Francisella*) and PagL (3-position deacylase from *Salmonella*) were ectopically expressed in the *Shigella* backgrounds. Targeted lipid A dephosphorylation (LpxE), deacylation (PagL), or execution of both modifications (Dual) was confirmed using MALDI-TOF MS. Lipid A dephosphorylation, rather than deacylation, effectively diminished LPS-induced pro-inflammatory immune signaling. Furthermore, deacylation combined with dephosphorylation did not further reduce LPS-mediated signaling. Due to this, only dephosphorylated lipid A mutants (LpxE-modified) were generated in WRSs2 and WRSf2G12, generating WRSs2E and WRSf2G12E. These strains had reduced LPS-mediated pro-inflammatory cytokine production *in vitro* and severely blunted endotoxemia *in vivo,* yet they remained as capable as their parental strains to invade epithelia and generate immunogenicity against their O-antigen. Altogether, we characterize two live-attenuated *Shigella* vaccine candidates (WRSs2E and WRSf2G12E), altered only in their lipid A region, which are greatly detoxified without any consequence to phenotypic traits suggesting that such live-attenuated strains are a promising approach to develop a safe and effective *Shigella* vaccine.

## Results

### Targeted lipid A modifications in *Shigella*

**Figure 1:**
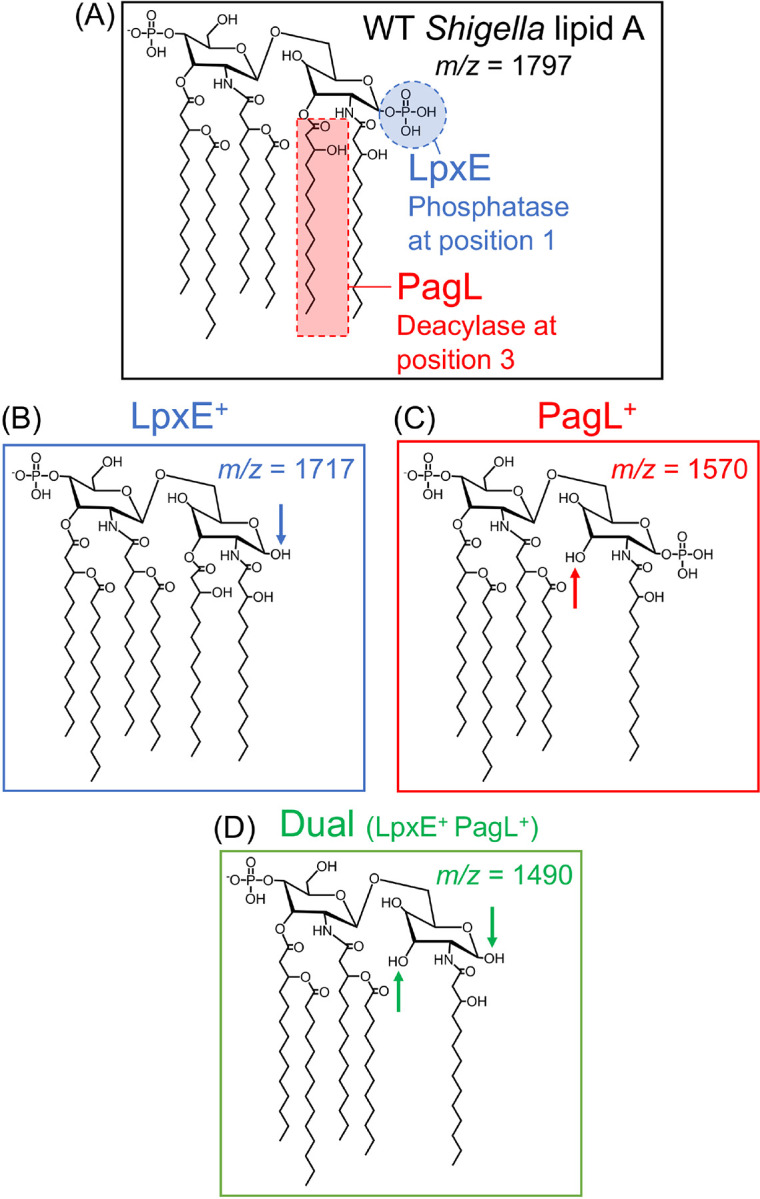
Lipid A modifications generated by BECC constructs used in this study (A) The WT lipid A structure of *Shigella* along with the (B) LpxE- (C) PagL- and (D) Dual-modified resultant structures, lacking a phosphate, 3OH C14 acyl chain, or both, respectively. The expected *m/z* for the [M-H]^−^ ions observed in MALDI-TOF spectra are displayed for each structure.

We initially utilized our BECC system to engineer targeted lipid A modifications (Fig 1) in *S. sonnei* Moseley and *S. flexneri* 2a 2457T, the wild-type (WT) strains upon which WRSs2 and WRSf2G12 were derived, respectively. Expression of the lipid A biosynthetic enzymes LpxE and PagL alone, or in combination (termed “Dual”), from the osmotically inducible pSEC10M plasmid resulted in targeted modification of the lipid A structure, which was confirmed via MALDI-TOF MS analysis (Fig S1A, B). Successful lipid A modification in WT strains prompted the expression of the individual enzymes in first-generation live-attenuated vaccine strains WRSS1 and SC602. Similar to the WT strains, upon plasmid-based expression of BECC constructs, lipid A dephosphorylation and deacylation were observed in WRSS1 and SC602 by MALDI-TOF MS, respectively (Fig S1C, D). This suggested that targeted lipid A modifications could be achieved in both WT and genetically attenuated strains of *Shigella*.

We next assessed whether these targeted lipid A modifications affected the immunostimulatory capacity of *Shigella* LPS. Purified LPS from Moseley and 2457T was normalized to keto-deoxy-octanoate (Kdo), a conserved sugar within the core oligosaccharide, and used to stimulate NF-*κ*B reporter cells. Stimulation with LpxE- and Dual-modified LPS resulted in a pronounced reduction in NF-*κ*B signaling compared to WT LPS, whereas stimulation with PagL-modified LPS generated comparable NF-*κ*B signaling to that of WT LPS (Fig S2). This suggested that lipid A dephosphorylation, rather than deacylation, was the most promising modification for reduced endotoxicity, and therefore, only LpxE- and Dual-modifications were pursued for the remainder of the study.

To remove the need for ectopic plasmid expression of the lipid A modification enzymes, we utilized Tn7 transposition to integrate the *lpxE* and Dual gene cassettes into the chromosome of Moseley and 2457T. Second-generation vaccine strains WRSs2 and WRSf2G12 were also chromosomally integrated with *lpxE*; however, the Dual gene cassette could not be chromosomally integrated into these specific vaccine strains. Chromosomally integrated strains were utilized for the remainder of the study. Moseley and 2457T are designated with LpxE^+^ or Dual to indicate what chromosomal integration they contain. Vaccine strains WRSs2 and WRSf2G12 chromosomally integrated with *lpxE* are designated as WRSs2E and WRSf2G12E, respectively.

**Figure 2:**
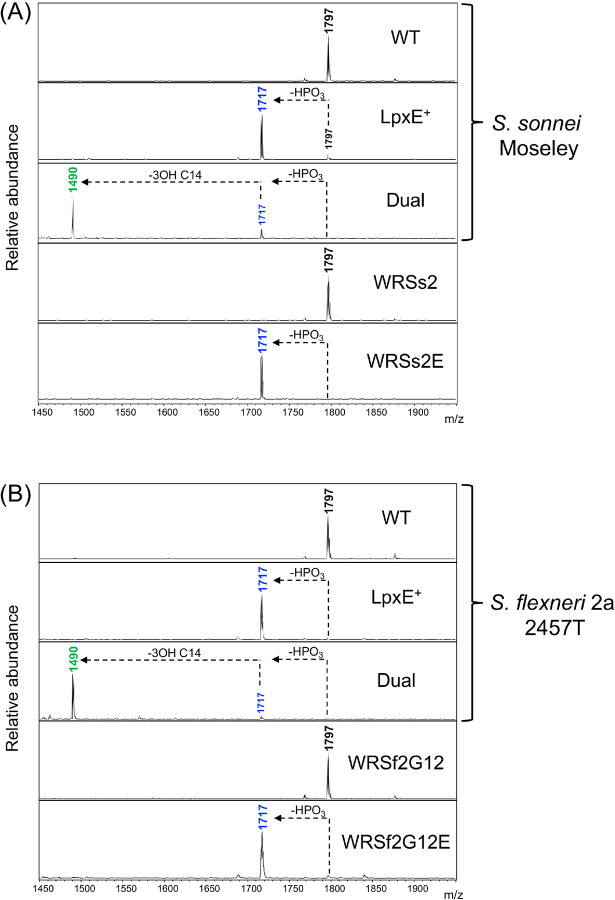
MALDI-TOF MS analysis of lipid A related peaks for BECC-modified *Shigella* strains Representative MALDI-TOF MS spectra for WT and vaccine strains of (A) *S. sonnei* and (B) *S. flexneri* 2a chromosomally expressing *lpxE* or Dual. Spectral peaks represent [M-H]^−^ ions. Colored peaks correspond to the expected structures detailed in Figure 1. Arrows depict the loss of a phosphate (HPO_3_) or acyl chain (3OH C14) from the indicated lipid A species.

Using MALDI-TOF MS, we confirmed that chromosomal expression of *lpxE* and Dual resulted in lipid A modification across the various *Shigella* backgrounds (Fig 2). Site-specificity of the modifications was confirmed via MALDI-TOF MS/MS using the FLAT^n^ approach^48^. WT, LpxE-, and Dual-modified LPS each synthesized their expected base peaks at *m/z* 1797, 1717, and 1490, respectively (Fig S3A), which were selected as the precursor ions for fragmentation (Fig S3B, circled in red). The large cluster of spectral peaks at *m/z* less than 1490 represents cardiolipin (Fig S3A), another phospholipid that resides in the outer leaflet along with lipid A. Spectral peaks representing fragments from the precursor ions (Fig S3B) were mapped to the proposed chemical structures of WT, LpxE-, and Dual-modified *Shigella* lipid A (Fig S3C). Determination of the location of the terminal phosphate modification at the 1-position was confirmed by the fragment ion still present at *m/z* 690 in the LpxE-modified structure, and the deacylation modification at the 3-position was confirmed by the ions generated from the ^0,2^A_2_ cross-ring cleavage event (Fig S3C).

### BECC-modified *Shigella* strains phenocopy their isogenic parental strains

Live-attenuated *Shigella* strains have fundamental requirements that enable them to function as effective oral vaccines. First, large-scale production requires efficient growth in culture. Secondly, these strains must be capable of invading gut epithelia, a step during infection that elicits the host immune response required for mucosal immunity^14^. Lastly, host defense responses must be unaltered, such as the secretion of CXCL8 from the gut epithelia, which recruits polymorphonuclear leukocytes to the site of infection to clear the bacteria^10^. Thus, any limitation with respect to growth, invasion, or the host response would render our BECC-modified *Shigella* strains unsuitable to be used as vaccine candidates.

To evaluate the capacity to grow in culture, we assayed the growth kinetics of our BECC-modified *Shigella* and compared it to their isogenic parental strains. Over the course of 15 hours, BECC-modified *Shigella* showed minimal growth alterations as compared to their unmodified counterparts (Figure S4), suggesting BECC-modification had minimal impact on the capacity of the *Shigella* strains to grow *in vitro*.

**Figure 3:**
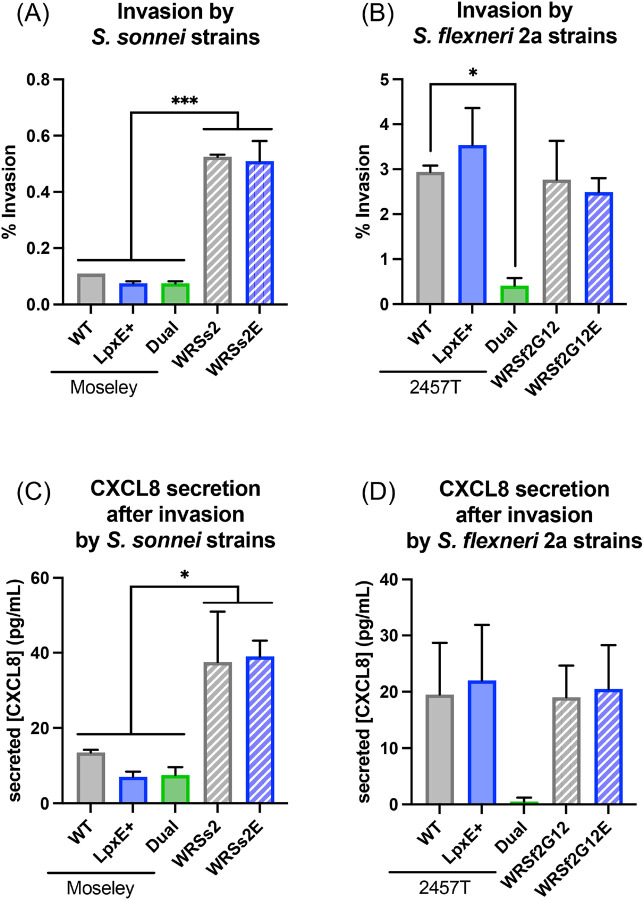
Invasion of epithelial cells by *Shigella* and corresponding CXCL8 production Invasion of HT29 cells after 4 hours of infection (MOI of 10) with (A) *S. sonnei* and (B) *S. flexneri* 2a strains. CXCL8 production in the cell supernatant after the 4-hour infection with (C) *S. sonnei* and (D) *S. flexneri* 2a strains. Statistical significance determined by ordinary one-way ANOVA. * and *** represent p-values of ≤ 0.05 and ≤ 0.001, respectively.

To evaluate the invasion of epithelia by *Shigella* (intracellular bacterial burden recovered as a percentage of the inoculum), we employed a gentamicin protection assay^26,28,29^. The BECC-modified *S. sonnei* strains invaded similarly to their parental strains. As found previously^26^, WRSs2 strains invaded the epithelial cells significantly more than the Moseley strains (Fig 3A). For *S. flexneri 2a* strains, while LpxE-modification did not impact invasiveness, Dual-modification resulted in a significantly lower invasion than the WT (Fig 3B), likely due to a high frequency of invasion plasmid loss compared to the other *S. flexneri* 2a strains (data not shown). Beyond invasion, we also measured CXCL8 concentrations in the supernatant to assess the host response to infection with our BECC-modified *Shigella,* which showed the same pattern as invasion (Figure 3C, D). This suggested that, except for the Dual-modification in 257T, the targeted lipid A modifications did not impact the phenotypic traits of *Shigella,* such as growth, invasion of gut epithelia, or the host response to infection.

### Lipid A modifications reduced endotoxicity *in vitro* and *in vivo*

**Figure 4:**
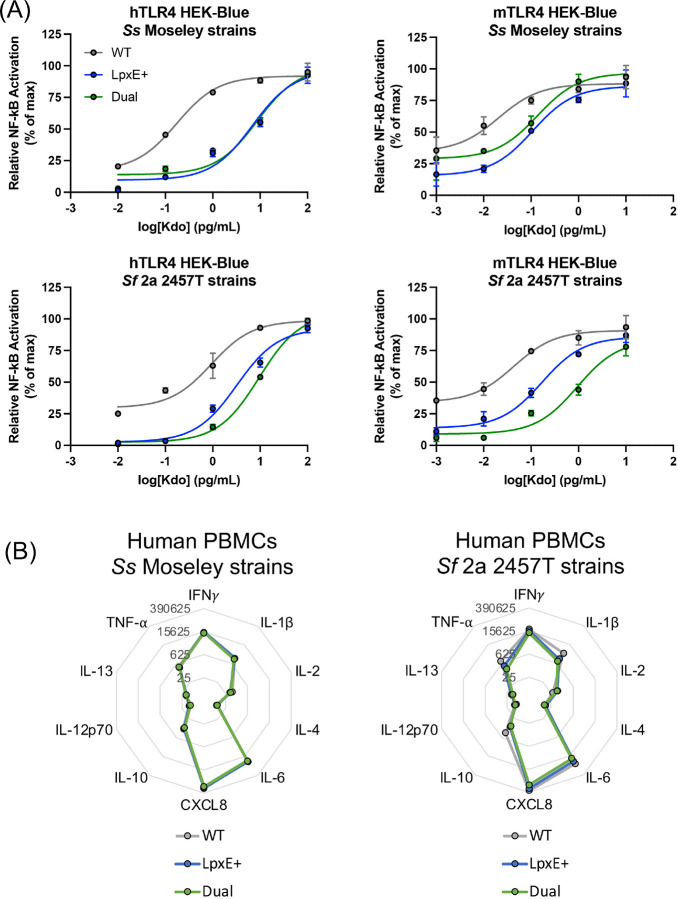
Stimulation of reporter and primary cells with Kdo normalized LPS from *Shigella* (A) HEK-Blue cells stably expressing an NF-*κ*B reporter under the control of the human and mouse orthologs of TLR4/MD-2/CD-14 (named hTLR4 or mTLR4, respectively) were stimulated across 10-fold dilutions, in duplicate, of Kdo standardized LPS for 18 hours at 37°C with 5% CO_2_. LPS was purified from *S. sonnei* Moseley or *S. flexneri* 2a 2457T. (B) Representative cytokine profile from one PBMC donor, as measured by MSD multiplex, upon stimulation of the PBMCs with the aforementioned LPS at 1 pg/mL Kdo for 48 hours at 37°C with 5% CO_2_. Numbers in grey denote cytokine concentration in pg/mL.

Since detoxification of *Shigella* lipid A was the primary objective of this study, we determined the level of endotoxicity using purified LPS from the chromosomally integrated strains. HEK-Blue cells stably expressing either the human or mouse orthologs of TLR4/MD-2/CD-14 (hereafter referred to as “hTLR4” or “mTLR4”) and containing an NF-*κ*B reporter were stimulated with Kdo normalized LPS from Moseley and 2457T. Stimulation with LpxE- and Dual-modified LPS resulted in diminished NF-*κ*B activation in both hTLR4 and mTLR4 reporter cell lines (Fig 4A). Similar results were observed in NF-*κ*B reporter cell lines that endogenously expressed the human (THP-1 Dual) or mouse (RAW-Blue) orthologs of TLR4 and its coreceptors (Fig S5). Furthermore, stimulation with LPS from WRSs2E and WRSf2G12E displayed reduced NF-*κ*B signaling compared to LPS from their respective parental strain (Fig S6). Altogether these data suggested that the potent pro-inflammatory cytokine production from WT *Shigella* LPS is greatly diminished upon LpxE-modification.

To examine the cytokine profile from primary cells, we stimulated human peripheral blood monocytes (PBMCs) from 4 different study participants with the same panel of purified LPS molecules and measured the cytokine and chemokine concentrations in the supernatant by multiplex analysis. BECC-modified LPS induced a similar cytokine profile to WT LPS with CXCL8, IL-6, IFN*γ*, IL-1β, TNF-α, and IL-10 present in decreasing abundance, respectively (Fig 4B). This pattern was conserved across the 4 participants and with LPS from WRSs2 and WRSf2G12 (Fig S7). Notably, production of these cytokines and chemokines was dampened upon stimulation with BECC-modified LPS, as compared to unmodified LPS from 2457T and WRSf2G12 (Fig S7A). Reduced cytokine and chemokine concentrations were comparable between LpxE- and Dual-modified 2457T LPS, which again suggested that dephosphorylation alone was sufficient to decrease pro-inflammatory cytokine production. Similarly, LpxE- and Dual-modified LPS from Moseley demonstrated reduced levels of cytokine and chemokine production; however, WT LPS from Moseley was less stimulatory than expected (Fig S7B). A separate experiment showed that LpxE- and Dual-modified LPS from Moseley demonstrated reduced induction of TNF-α from PBMCs, and to a similar degree, compared to WT LPS (Fig S7C), which confirmed that dephosphorylated *Shigella* lipid A does indeed reduce pro-inflammatory cytokine production *in vitro* across all *Shigella* backgrounds.

**Figure 5:**
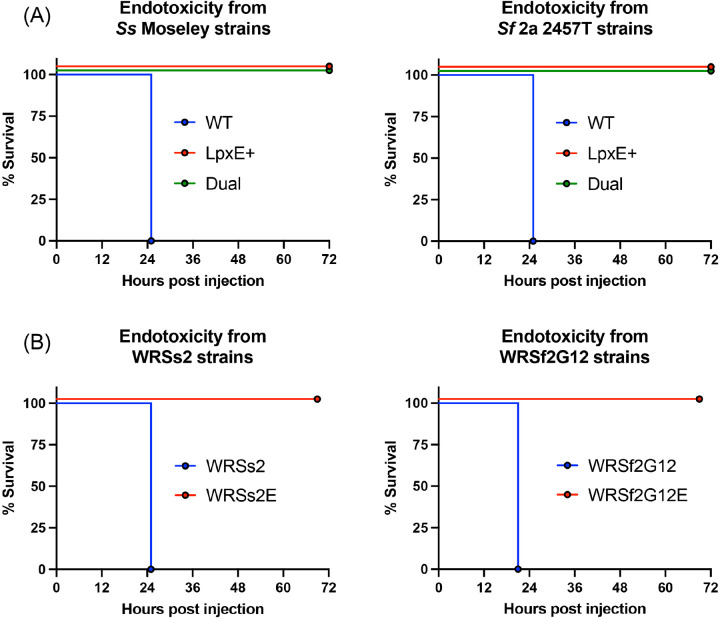
Assessment of the toxicity of *Shigella* LPS via a murine acute endotoxemia model Survival curves for mice (n=5) receiving a Kdo normalized dose of LPS intraperitoneally, representative of 15 mg/kg, using purified LPS from (A) WT strains and (B) vaccine strains of *S. sonnei* and *S. flexneri* 2a.

To confirm that the BECC-modified LPS was also detoxified *in vivo*, we employed an acute murine endotoxicity study whereby a lethal dose of LPS was injected intraperitoneally into mice, and their health status was monitored over the course of 72 hours. Whereas injection of WT LPS from Moseley or 2457T was lethal by 24 hours post-injection, all mice receiving LpxE- or Dual-modified LPS survived after receiving the same dose (Fig 5A). The same pattern was observed using LPS from WRSs2 and WRSf2G12 (Fig 5B). Altogether, this data suggested that dephosphorylation of lipid A was sufficient to reduce endotoxicity *in vivo*.

### LpxE-modification did not compromise the immunogenicity of the *Shigella* vaccine strains

**Figure 6:**
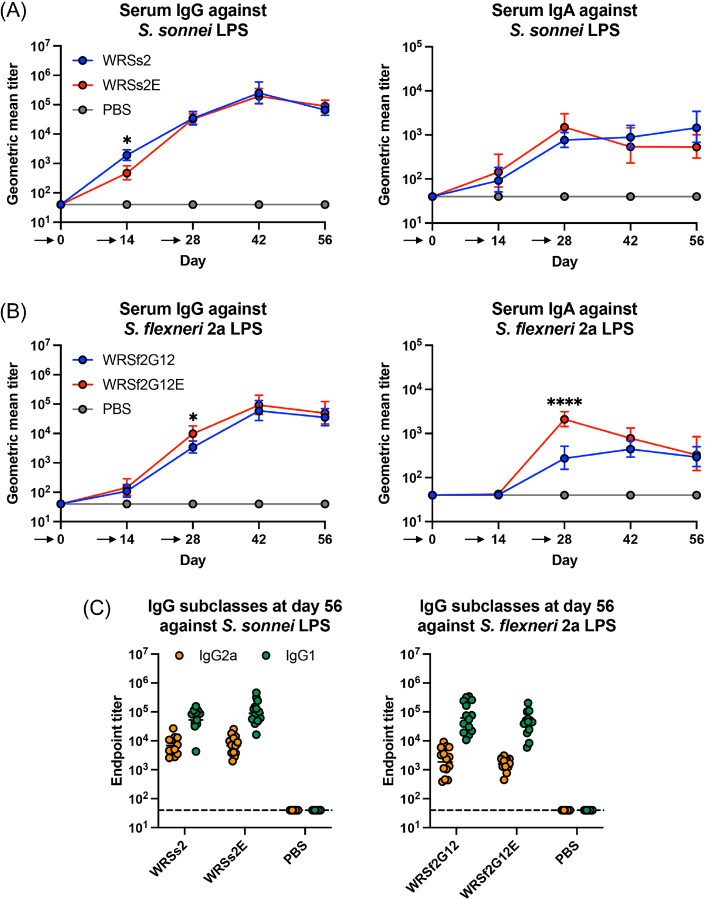
Antibody titers from a *Shigella* murine vaccine study Serum IgG and IgA geometric mean titers against (A) *S. sonnei* Moseley LPS or (B) *S. flexneri* 2a 2457T LPS for mice (n=15) vaccinated intranasally with 10^6^ CFU at day 0, 14, and 28 as indicated by the arrows below the X-axis. (C) Serum IgG2a and IgG1 titers at day 56 against serotype-specific LPS. Statistical significance was determined by 2way ANOVA. * and **** represent p-values of ≤ 0.05 and ≤ 0.0001, respectively.

To evaluate their potential use as vaccine candidates, we compared the immunological response of WRSs2E and WRSf2G12E to their parental strains in a mouse model. As mice do not experience diarrheal episodes from ingestion of *Shigella*, we evaluated vaccine efficacy through the generation of *Shigella*-specific immunological responses. Using three routes of vaccination (oral, intranasal, and intramuscular) and two different types of vaccines (live bacteria or purified LPS) (Fig S8A), we determined that intranasal administration of live bacteria at Day 0, 14, and 28 (prime-boost-boost, respectively) generated the most reliable serum antibody response against serotype-specific LPS (Fig S8B). Using this intranasal approach and the same vaccination scheme of prime-boost-boost at Day 0, 14, and 28, respectively, we showed that vaccination with WRSs2E or WRSf2G12E elicited strong serum IgG and IgA responses against serotype-specific LPS, that mimicked the response from their parental strains (Fig 6A, B). Although statistically significant differences in antibody titers were observed between the parental and LpxE-modified strains at specific time points, these differences did not remain throughout the entire vaccine study. At Day 56, four weeks after the final vaccine dose was delivered, the skewing of IgG subclasses 2a and 1 was similar for WRSs2E and WRSf2G12E compared to their parental strains (Fig 6C). The same patterns were observed in a second independent vaccine study (Fig S9). Ultimately, this data supports the notion that WRSs2E and WRSf2G12E promote a similar adaptive immune response as their parental strains.

## Discussion

Despite significant advances in our understanding of *Shigella* pathogenesis and the development of many vaccine candidates^49^, to date, there is no FDA-licensed vaccine available. This study utilized second-generation vaccine candidates WRSs2 and WRSf2G12, whose first-generation strains were generally well tolerated in clinical trials but deemed too reactogenic to be considered safe for general use^33,37^. These second-generation vaccine strains contain a suite of genetic manipulations that remove known enterotoxins and reduce the spread of the bacterium across gut epithelia^24,26,29^. In this study, we describe the engineering and characterization of an additional modification, specifically the dephosphorylation of their lipid A structure, to generate WRSs2E and WRSf2G12E, which have reduced endotoxicity while retaining the same phenotypic traits as their isogenic parental strains.

The lipid A modifications engineered in this study utilized the BECC approach whereby prior identification of lipid A modifying enzymes from a variety of Gram-negative bacteria then enabled the expression of select enzymes within a bacterium of interest. Previously, the BECC system has been employed to generate custom-designed lipid A molecules in *Yersinia*^50–56^; however, this study extends its use to *Shigella* species, suggesting it is applicable to a variety of Gram-negative bacteria. More specifically, we showed that BECC enabled robust lipid A modification in both WT and vaccine strains of *Shigella*. Whereas multiple lipid A related spectral peaks were present upon plasmid-based expression of BECC constructs (Fig S1), chromosomal expression generated a single spectral peak (Fig 2) indicative of more complete lipid A modification on the outer membrane. This demonstrates a newfound approach for the expression of BECC enzymes since here we showed that chromosomally expressing strains are both stable and robustly modify their lipid A, all without the requirement for antibiotic selection.

Furthermore, to function as an effective oral vaccine, specific phenotypic requirements are required that may be altered upon engineering modified lipid A strains. We showed that dephosphorylation of lipid A had no impact on invasion or growth; however, Dual-modification (both dephosphorylation and deacylation) caused *S. flexneri* 2a 2457T to lose its invasion plasmid more frequently, likely a consequence of increased membrane stress. This is further supported by the inability to chromosomally integrate the Dual construct into WRSs2 or WRSf2G12, suggesting a limit to the degree to which lipid A can be modified in already genetically attenuated strains.

Additionally, we showed that dephosphorylation at the 1-position via LpxE was sufficient to effectively blunt the LPS-induced pro-inflammatory response from both human and murine immune cells. This is analogous to other contexts, such as sepsis, where human alkaline phosphatase dephosphorylates LPS to reduce inflammatory signaling^57^. Traditionally, however, it is thought that deacylation of lipid A reduces LPS-induced signaling through TLR4/MD-2 as tetra- and penta-acylated structures are generally less immunostimulatory than hexa-acylated^42^. Here, we showed that PagL-mediated penta-acylated *Shigella* lipid A, lacking the 3-position backbone acyl chain, did not reduce LPS-mediated signaling (Fig S2). It has been shown that penta-acylated *Shigella* lipid A lacking an acyl chain at a different site has reduced LPS-induced signaling. Rossi *et. al*., showed that an *htrB* mutant in *S. sonnei*, whose lipid A lacks the secondary acyl chain at the 2’-position, displayed reduced TLR4 signaling in NF-*κ*B reporter cells; however, the same mutation in *S. flexneri* 2a led to a compensatory C16:1 addition and no reduction in signaling compared to WT LPS^46^. This suggests that detoxification of *Shigella* lipid A via deacylation is site-specific. Furthermore, it emphasizes the complexity of achieving complete lipid A deacylation via genetic manipulation in *Shigella*. For instance, *Shigella* contains two *msbB* genes (both encoding MsbB/LpxM), one chromosomal and one on its invasion plasmid^58,59^. Additionally, *Shigella* can induce *lpxP* (encodes LpxP, C16:1 acylase) in the absence of *htrB (*encodes HtrB/LpxL) under stress-inducing conditions^45,60,61^. Altogether, this genetic redundancy of lipid A biosynthetic enzymes in *Shigella* highlights its drive to maintain hexa-acylated lipid A. Using BECC avoids this complication, as it introduces exogenous lipid A modifying enzymes and prevents induction of compensatory mechanisms that revert its lipid A back to the hexa-acylated state. This is emphasized in the present study as primarily a single spectral peak was observed in the MALDI-TOF MS spectra upon expression of BECC constructs across the various *Shigella* backgrounds (Fig 2), suggesting that the outer membrane contains predominantly the targeted lipid A structure without any compensatory lipid A modifications.

Despite remodeling of the lipid A region of LPS in the WRSs2E and WRSf2G12E, murine vaccination with these strains generated similar immune responses to their isogenic parental strains (Fig 6 and S9). This suggested that serotype-specific immunity in response to infection with these strains was unaffected by BECC modification. Since *Shigella* does not cause diarrheal disease upon ingestion in rodents, only immunological responses were evaluated in this study; however, the first-generation variants of WRSs2 (WRSS1) and WRSf2G12 (SC602) have shown efficacy against shigellosis in humans^22,36^. Since LpxE-modification abrogates the toxic effects of LPS and does not appear to impact immunogenicity, this supports the notion that oral vaccination in humans with WRSs2E and WRSf2G12E would have reduced reactogenicity while maintaining robust immunogenicity and protection against shigellosis.

Overall, the need for a *Shigella* vaccine remains a priority. While it has been proposed that endemic *Shigella* can be controlled via public health efforts, the low infectious dose combined with its capacity to acquire extreme drug resistance has bolstered vaccination as a promising option to control the spread of this pathogen. To date, live-attenuated *Shigella* vaccines have shown immunological success in humans and, in some cases, protection against virulent challenges; this suggests that optimization of current live-attenuated vaccine candidates is a promising approach. A significant drawback with live-attenuated vaccine candidates, however, is the adverse effects from ingestion of high doses of bacteria containing the immunostimulatory hexa-acylated *Shigella* LPS. The BECC-modified vaccine strains of *Shigella* developed in this study, namely WRSs2E and WRSf2G12E, contain detoxified LPS and thus have promise to be better tolerated, safer, live-attenuated vaccine candidates.

## Methods

### Ethics statement

All animal procedures were approved by the University of Maryland, Baltimore Institutional Animal Care and Use Committee (IACUC #0222002). In all studies, female BALB/cJ mice (Jax Laboratories) were utilized. Mouse husbandry was conducted according to the procedures established at the University of Maryland, Baltimore.

### Bacterial strains and growth conditions

Bacterial strains used in these studies are listed in Table S1. Bacteria were grown at 30°C or 37°C in Lysogenic Broth (Teknova) and Tryptic Soy broth (TSB) or on Tryptic Soy agar (TSA) (Becton Dickinson) supplemented with 50 μg/mL neomycin (Sigma) or 60 μg/mL carbenicillin (Sigma) as needed. All strains were supplemented with 1 mM MgCl_2_ to repress the PhoPQ two-component regulatory system. Growth curves were performed in a flat-bottom 96-well uncoated sterile plate (Costar) and recorded using a Cerillo Stratus instrument (Cerillo). Each well was inoculated with 10^5^ CFU in 200 μL TSB and incubated with shaking (180 RPM), at 37°C for 15 hours. Absorbance readings at 600 nm were taken every 15 minutes.

### Molecular genetic techniques

Standard DNA techniques, liquid media, and agar plates were used as described^62^. Restriction endonucleases and T4 DNA ligase were used as recommended by the manufacturer (New England Biolabs). DNA used for cloning purposes was PCR amplified using 10mM dNTP mix (Thermo Scientific) and high-fidelity DNA polymerases Q5 (New England Biolabs) or Pfu ultra II fusion HS (Agilent) according to manufacturer’s instructions. Go-Taq polymerase (Promega) was used for genetic screening. DNA oligonucleotides were obtained from Integrated DNA Technologies and are listed in Table S2. All plasmid constructs (Table S3) were confirmed by double-stranded sequencing (Azenta) and maintained in *E. coli* DH5α or *E. coli* TOP10 (ThermoFisher).

### Generation of plasmid-based BECC-modified *Shigella* strains

LPS modifying enzymes LpxE, PagL, and LpxE-PagL in tandem (termed “Dual”) were first cloned and expressed in pSEC10^63^ under the osmotically controlled *E. coli ompC* promoter (P_*ompC*_). A codon-optimized form of *lpxE* from *Francisella novicida* was synthesized by GenScript and cloned into pUC57, yielding pUC57::*lpxE*. The 720 bp *lpxE* gene was amplified by PCR from pUC57::*lpxE* using Q5 polymerase (New England Biolabs) and primer set *lpxE*-F/*lpxE*-R, trimmed with restriction enzymes BamHI and NheI, and ligated into the BamHI/NheI site of pSEC10 resulting in the construct pSEC10::P_*ompC*_-*lpxE*. The *pagL* gene was amplified by PCR from *Salmonella minnesota* (Genbank accession AE006468.2) using Q5 polymerase and primer set *pagL*-F/*pagL*-R. A 570 bp amplicon was trimmed with BamHI/NheI and ligated into the 6630 bp BamHI/NheI digested fragment of pSEC10 yielding pSEC10::P_*ompC*_-*pagL*. Both *lpxE* and *pagL*, each preceded by a ribosomal binding site, were synthesized in tandem behind P_*ompC*_ and cloned into vector pUC57K by GenScript yielding pUC57K::P_*ompC*_-Dual. The initial subcloning of *pagL* into pSEC10 included six additional bps (GTGTAT) that encoded an alternative start codon present in the *S. minnesota* sequence; this 6 bp sequence was not included in the Dual construct. The P_*ompC*_-Dual gene cassette was then cloned into a modified version of pSEC10 (pSEC10M). Briefly, primer set pSEC10M-F/pSEC10M-R was self-annealed, trimmed with EcoRI/NheI, and ligated into a 5775 bp gel purified EcoRI/NheI digested pSEC10 and transformed into *E. coli* TOP10. The resulting pSEC10M had a multiple cloning site [EcoRI-NotI-SwaI-NheI] in place of the *ompC* promoter and *clyA* gene. The 1.9 kb SwaI P_*ompC*_-Dual gene cassette isolated from pUC57K::P_*ompC*_-Dual was ligated into the SwaI digested site of pSEC10M and transformed into *E. coli* TOP10 yielding construct pSEC10M::P_*ompC*_-Dual. Plasmids pSEC10::P_*ompC*_-*lpxE*, pSEC10::P_ompC_-*pagL*, and pSEC10M::P_ompC_-Dual were electroporated into the wild-type strains of *S. sonnei* and *S. flexneri* 2a and selected on TSA with neomycin. Successful transformants were used to inoculate a 2 mL overnight culture in TSB with neomycin, which received a final concentration of 15% glycerol, followed by storage at −80°C.

### Generation of *attTn7* chromosomally integrated BECC-modified *Shigella* strains

We mobilized P_*ompC*_-*lpx*E and P_*ompC*_ -Dual into the *Shigella* chromosome *attTn7* site using a site-specific insertion method utilizing the Tn7 recombination machinery on a temperature-sensitive plasmid pGRG36^64^. A 1225 bp amplicon of the P_*ompC*_-*lpxE* gene cassette was generated using template pSEC10::P_*ompC*_-*lpxE*, Q5 polymerase (New England Biolabs) and primer set P_*ompC*_-F/*lpxE*-R. This was then blunt-ligated into the SmaI digested site of pGRG36, yielding pGRG36::P_*omp*_*C*-*lpxE*. A 1.9 kb P_*ompC*_-Dual gene cassette flanked by SwaI restriction sites was isolated from pUC57K::P_*ompC*_-Dual and ligated into the SmaI site of pGRG36 yielding pGRG36::P_*ompC*_-Dual. The resulting pGRG36 construct was transformed into *E. coli* S17–1 and introduced into wild-type and 2^nd^ generation vaccine strains of *S. sonnei* and *S. flexneri* 2a by conjugal mating. Briefly, 2 mL cultures were grown overnight. *E. coli* S17–1 plasmid transformants were grown in the presence of carbenicillin at 30°C whereas *Shigella* cultures were grown without antibiotic at 37°C. Filter matings were performed by mixing 100 μL of *Shigella* with 50 μL of *E. coli* S17–1 plasmid transformants, concentrated by centrifugation (8k × g for 1 minute), resuspended in 200 μl TSB, spread onto a 0.45 μM nylon filter (MSI Magna nylon 66) placed on the center of a TSA plate, and incubated for 5 hours at 30°C. Bacteria on the nylon filter were then resuspended in 1 mL TSB and plated onto TSA containing 0.01% Congo red dye (Sigma- Aldrich), carbenicillin, and 1 mM MgCl_2_ and incubated overnight at 30°C. *Shigella* conjugants that grew at 30°C and were carbenicillin resistant were screened by PCR for the presence of *lpxE* and the *Shigella* invasion plasmid using GoTaq (Promega), and primer sets *lpxE*-F/*lpxE*-R (*lpxE*) and *ospD3*-F/*ospD3*-R (*ospD3*) or *lpxE*-F/*lpxE*-R (*lpxE*) and *ipaB*-F/*ipaB*-R (*ipaB*) for WT and vaccine strains, respectively. Bacteria were plated on TSA containing 0.1% arabinose and incubated at 42°C overnight to promote Tn7 recombination and simultaneous curing of pGRG36. Isolates that were carbenicillin sensitive and Congo Red positive, were assayed by PCR for the presence of *lpxE* using primer set *lpxE*-F/*lpxE*-R and for the *Shigella* invasion plasmid using ospD3 primer set *ospD3*-F/*ospD3*-R or *ipaB* primer set *ipaB*-F/*ipaB*-R for WT and vaccine strains, respectively. Confirmed integrants were stored in 15% glycerol at −80°C. Bacterial genomes were sequenced at the Microbial Genome Sequencing Center (SeqCenter) and analyzed using the RAST software^65^ which confirmed the insertion of the gene cassettes into the *attTn7* site.

### MALDI-TOF MS and MS/MS analysis of lipid A

Functional screening of *Shigella* strains to confirm lipid A modification was performed using the Fast Lipid Analysis Technique (FLAT) coupled to MALDI-TOF MS analysis^66^. Briefly, a single colony was spotted on a MALDI plate and overlaid with 1 μL of citrate buffer (200 mM citric acid, 100 mM trisodium citrate, pH 3.5). The plate was incubated in a humidified, closed, glass chamber for 30 minutes at 110°C, cooled, washed with endotoxin-free water, and 1 μL of 10 mg/mL norharmane matrix (Sigma-Aldrich) dissolved in chloroform : methanol (2:1 v/v) was spotted onto the samples on the MALDI plate. MALDI-TOF MS analysis was performed using a Bruker Microflex LRF equipped with a 337 nm nitrogen laser. Spectra were acquired in the negative ion and reflectron mode. Analyses were conducted at < 60% global intensity with 300 laser shots for each spectrum acquisition. Spectra were recorded in triplicate. Agilent ESI tune mix (Agilent) was used for mass calibration. FlexAnalysis software version 3.4 (Bruker) was used to process the mass spectra with smoothed and baseline corrections. Further structural lipid A characterization was conducted by tandem mass spectrometry (MS/MS) analysis using the FLAT^n^ procedure^48^. The FLAT process above was repeated, except the colony spotted onto an indium tin oxide (ITO) slide instead of a MALDI plate. MS/MS analysis was performed using a Bruker MALDI trapped ion mobility spectrometry Time-of-Flight (timsTOF) mass spectrometer equipped with a dual ESI/MALDI source with a SmartBeam 3D 10 KHz frequency tripled 355 nm Nd:YAG laser. The system was operated in “qTOF” mode (tims deactivated). Ion transfer tuning used the following parameters: Funnel 1 RF: 440.0 Vpp, Funnel 2 RF: 490.0 Vpp, Multipole RF 490.0 Vpp, is CID Energy: 0.0 eV, and Deflection Delta: −60.0 V. The quadrupole used the following values for MS mode: Ion Energy: 4.0 eV and Low Mass 700.00 m/z. Collision cell activation of ions used the following values for MS mode: Collision Energy: 9.0 eV and Collision RF: 3900.0 Vpp. The precursor ion was chosen by inputting targeted *m/z* values including two digits beyond the decimal point. Typical isolation width and collision energy were set to 4 – 6 *m/z* and 100 – 110 eV, respectively. Focus Pre-TOF used the following values: Transfer time 110.0 μs and Pre pulse storage 9.0 μs. Agilent ESI Tune Mix (Agilent) was used to perform calibration. MALDI parameters in qTOF mode were optimized to maximize intensity by tuning ion optics, laser intensity, and laser focus. All spectra were collected at a laser diameter of 104 μm with beam scan on using 800 laser shots per spot using either 70% or 80% laser power. MS/MS data were collected in negative ion mode. In all cases, a matrix of 10 mg/mL norharmane dissolved in chloroform : methanol (2:1 v/v) was used. mMass software version 5.5.0^67^ was used to process the mass spectra with smoothed and baseline corrections. Identification of all fragment ions were determined based on ChemDraw Ultra version 10.0.

### LPS extraction and purification

LPS was isolated using the double hot phenol method. Briefly, two liters of bacterial culture were harvested by centrifugation and resuspended in 90% phenol : endotoxin-free water (1:1 v/v) and incubated at 65°C for 1 hour. After centrifugation, the aqueous phase was isolated from the two-phase solution (repeated three times total and pooled) and dialyzed for 36 hours against deionized water using pre-treated 1 kD MWCO RC tubing (Spectrumlabs.com), followed by flash freezing and lyophilization. The lyophilized product was resuspended in 20 mM Tris-HCl pH 8.4 supplemented with 2 mM MgCl_2_ and digested using 500 units of Benzonase and 100 μg/mL Dnase I for 2 hours at 37°C. The pH was subsequently adjusted to 7.4 using 1 N HCl and the solution further digested with 100 μg/mL Proteinase K for 2 hours at 37°C. Water-saturated phenol was added, vortexed, and centrifuged (8,000 × g), and the upper aqueous phase was collected, dialyzed, and lyophilized. Further isolation of LPS was performed by serial washes in chloroform : methanol (2:1 v/v) as described^68^. The LPS was separated from contaminating lipoproteins as described^69^ by resuspension in 0.2% TEA (triethylamine) and 0.5% DOC (deoxycholate), followed by the addition of 37°C water-saturated phenol and the upper aqueous phase collected. Finally, the LPS product was precipitated by the addition of cold 100% ethanol and 30 mM sodium acetate followed by incubation for 18 hours at −20°C. The LPS precipitate was harvested by centrifugation (5,000 × g, 20 minutes), washed in cold 100% ethanol, resuspended in endotoxin-free water (Quality Biological), and lyophilized.

### Kdo assay for LPS quantification

2-keto-3-deoxyoctonate (Kdo) standards ranging from 12 – 48 μg/mL in endotoxin-free water and 1 mg/mL LPS solution solutions were hydrolyzed in 0.018 N sulfuric acid (H_2_SO_4_) at 100°C for 20 minutes, followed by the addition of 25 μL of 9.1 mg/mL periodic acid (H_5_IO_6_) in 0.125 N H_2_SO_4_ and incubation in the dark for 20 minutes. Samples then received 50 μL of 2.6% sodium arsenite (NaAsO2) in 0.5 N HCl was followed by the addition of 250 μL of 0.3% thiobarbituric acid (TBA). Samples were heated at 100°C for 10 minutes, quickly followed by the addition of 125 μL of dimethyl sulfoxide (DMSO), and the measurement of absorbance at 550 nm. The absolute quantification is based on the interpolation of the standard curve provided by the Kdo_2_ quantity. Half of the Kdo_2_ quantity, representing Kdo_1_ (referred to as simply “Kdo” in this study), was utilized for normalization.

### Murine acute endotoxemia

LPS solutions of 45 μg/mL Kdo_2_ (representative of 15 mg/kg if using dry weight instead) were prepared in sterile, endotoxin-free PBS (Quality Biology). LPS solutions were transferred to arbitrarily labeled tubes by a third-party observer to ensure blinding to group identifications and avoid bias in clinical score designations. Each mice received 100 μL of LPS solution using a slip tip 1 mL syringe attached to a 27-gauge ½ inch needle (Becton Dickinson) via the intraperitoneal route. Mice were monitored for 72 hours post-injection, receiving a clinical score/mouse based on appearance and mobility as described in Table S4. A clinical score of 5 required euthanization as a consequence of no movement, noticeable stress, and an inability to return upright if placed on their side.

### Cell culture media and conditions

RPMI-1640 (Gibco) complemented with 25 mM HEPES, 2 mM glutamine, 10% FBS, and 1% penicillin-streptomycin, referred to as cRPMI, was filter sterilized through a 0.22 μM filter flask and used for the THP-1 NF-*κ*B-SEAP reporter cell line (THP-1 Dual, Invitrogen). DMEM (Corning) complemented with 3.7 g/L sodium bicarbonate, 2 mM glutamine, 10% FBS, and 1% penicillin-streptomycin, referred to as cDMEM, was filter sterilized through a 0.22 μM filter flask and used for the HT29 cells (courtesy of Dr. Eileen Barry, UMB), mTLR4/hTLR4 HEK-Blue cells (Invitrogen), and RAW-Blue cells (Invitrogen). All cells were maintained at 37°C with 5% CO_2_.

### NF-*κ*B reporter cell line stimulations

Sterile, cell culture-treated, 96-well flat bottom plates (Costar) were seeded with HEK-Blue, RAW-Blue, or THP-1 Dual reporter cells at 6 × 10^4^ cells/well. THP-1 cells received 100 ng/mL of vitamin D_3_ (Sigma) prior to seeding in wells to enable cell differentiation. Cells were incubated for 18 hours at 37°C with 5% CO_2_, except the THP-1 cells, which were incubated for 72 hours to enable differentiation into monocyte-derived macrophages. Five 10-fold dilutions of Kdo standardized LPS ranging from 10^2^ pg/mL to 10^−2^ pg/mL was used to stimulate NF-kB production in cells. Cells were incubated at 37°C with 5% CO_2_ for 18 hours. Detection of NF-kB was quantified using the Quanti-Blue (QB) reagent prepared according to the manufacturer’s protocols (Invitrogen). The percent of relative NF-*κ*B activation was normalized to the maximum OD 630 nm measured. Points were plotted as the mean ± standard deviation of the relative NF-*κ*B activation at each concentration using GraphPad Prism version 9 and fitted using a nonlinear regression of the log(agonist) versus response (three parameters).

### Stimulation of primary peripheral blood monocytes

Human peripheral blood was collected from healthy adult study participants 18–40 years of age per a Boston Children’s IRB-approved protocol (protocol number X07-05-0223). All participants signed an informed consent form prior to enrollment. Heparinized whole blood was centrifuged (500 × g, 10 minutes) prior to removal of the upper layer of platelet-rich plasma. The plasma was centrifuged (3,000 × g, 10 minutes) and platelet-poor plasma (PPP) was collected and stored on ice. The remaining blood was reconstituted to its original volume with heparinized Dulbecco’s PBS and layered on Ficoll-Paque gradients (Cytiva) in Accuspin tubes (Sigma-Aldrich).

PBMCs were collected after centrifugation, washed twice with PBS, and seeded at 2 × 10^5^ cells/well in 96-well U-bottom plates (Corning) in RPMI-1640 media (Gibco) supplemented with 10% autologous PPP, 100 IU/ mL penicillin, 100 μg/mL streptomycin, and 2 mM L-glutamine. PBMCs were incubated with 1 pg/mL Kdo standardized LPS for 24 hours at 37 °C with 5% CO2. The supernatants were recovered after centrifugation (500 × g, 5 minutes) and analyzed for TNF-α quantification.

For multiplex analysis, frozen PBMCs from 4 independent human donors were instead obtained from AllCells, snap-thawed, washed twice with warm cRPMI, and seeded at 5 × 10^5^ cells/well in 96-well, sterile, uncoated U-bottom plates (Costar). PBMCs were incubated with 1 pg/mL Kdo standardized LPS for 48 hours at 37 °C with 5% CO2. The supernatants were recovered after centrifugation (400 × g, 5 minutes) and stored at −20°C until analyzed by MSD multiplex.

### Invasion assays

HT29 cells were seeded at a density of 5 × 10^5^ cells/well in 24-well flat bottom sterile plates (Corning) and incubated at 37°C for 18 hours with 5% CO_2_. *Shigella* cultures from overnight growth on TSA containing 0.01% Congo Red were used to generate a resuspension in sterile PBS pH 7.4. Inoculums of 5 × 10^6^ CFU were used to infect, giving an MOI of 10. Enumeration of the inoculation was confirmed in duplicate by plate counts on TSA. Bacteria were added to media-free, PBS-washed cell monolayers and the plates were centrifuged (3,000 × g, 5 minutes). After incubation for 90 minutes at 37°C with 5% CO_2_, cell monolayers were twice washed with sterile PBS, followed by the addition of cDMEM containing 50 μg/mL gentamycin (Sigma-Aldrich) and incubation for 2.5 hours. Supernatants were isolated and processed for human CXCL8 secretion by cytokine ELISA. The cell monolayer was twice washed with sterile PBS and lysed with 1% Triton X-100 (Sigma-Aldrich) in sterile PBS for 10 minutes at room temperature. Serial dilutions were plated in duplicate on TSA and incubated overnight at 37°C. The percentage of invasion was determined as the CFU/mL recovered normalized to the CFU/mL inoculated.

### Cytokine ELISA

Cytokine analysis of host cell culture supernatants was performed using DuoSet ELISA kits (R&D Systems) according to the manufacturer’s protocol. Briefly, plates were coated overnight at 4°C by adding 100 μL/well of 2 μg/mL capture antibody in ELISA coating buffer, washed three times PBS + 0.02% Tween-20 (PBST) and blocked with 300 μL/well 1% BSA in PBS for 1 hour at room temperature followed by three washes with PBST. Cell culture supernatants were diluted to reach a signal within the dynamic range. Bound cytokines were labeled by adding biotin-conjugated antibodies in block buffer (100 μL/well of 2 pg/mL) and incubated at room temperature for 2 hours. Plates were washed with PBST and incubated for 20 minutes with secondary antibody streptavidin-HRP, followed by the addition of a color substrate. The plates were read at both 450 nm and 562 nm and the difference taken as the final reading. The amount of cytokine is reported as picograms per mL of cell culture supernatant.

### Murine vaccination with live-attenuated *Shigella*

*Shigella* vaccine strains were grown at 37°C overnight on TSA containing 0.1% Congo Red. For intranasal vaccination, inoculums of 3.33 × 10^8^ CFU/mL and 3.33 × 10^7^ CFU/mL of *Shigella* were prepared in sterile PBS and kept at room temperature. Mice were anesthetized using a Matrx VIP 3000 vaporizer (Midmark Animal Health) with isoflurane (Fluriso, VetOne): oxygen (Airgas, OX USPEAWBDS) mixture (1:1 mixing) for 1–2 minutes and 15 μL of the vaccine inoculum was delivered to each nare (30 μL in total) using a pipette. For oral gastric vaccination, inoculums of 1 × 10^8^ CFU/mL and 1 × 10^7^ CFU/mL of *Shigella* were prepared in sterile PBS and kept at room temperature. Inoculums of 100 μL were delivered by intra-gastric gavage using a 2-inch-long plastic feeding needle (VWR) connected to a 1 mL syringe (Becton Dickinson). For both vaccination methods, mice were monitored for adverse reactions post-immunization. Enumeration of the vaccine inoculums was determined by plate counts on TSA.

### Murine vaccination with purified LPS

Purified LPS from wild-type *S. sonnei* Moseley was obtained as described above (see LPS extraction and purification). For internasal vaccination, solutions containing purified LPS at 1 mg/mL and 0.66 mg/mL, dissolved in sterile PBS, were delivered intranasally as described above. For intramuscular vaccination, solutions containing purified LPS at 0.6 mg/mL and 0.4 mg/mL, dissolved in sterile PBS, were prepared and stored at room temperature. Mice were immobilized using a restrainer, and 50 μL of the solution was injected using a 1 mL syringe (Becton Dickinson) into the caudal muscle after disinfecting the area with 70% ethanol. For both vaccination methods, mice were monitored for adverse reactions post-immunization.

### Sera collection

Mice were bled via the lateral saphenous vein using petroleum jelly and 27-gauge needles. Blood was collected in a microvette 200 Z-gel tubes (Sarstedt), and the sera were isolated by centrifugation (10,000 × g, 3 minutes) and stored in sealed uncoated 96-well flat bottom plates (Thermo Fischer) at −20°C.

### Enzyme-linked immunosorbent assay (ELISA)

Coating antigens used in ELISAs included purified LPS from wild-type *S. sonnei* Moseley or *S. flexneri* 2a 2457T. Nunc MaxiSorp plates (ThermoFischer) were coated with 5 μg/mL serotype-specific LPS in 100 mM carbonate coating buffer pH 9.6 (sodium bicarbonate/carbonate) and incubated for 3 hours at 37°C. Plates were washed with PBS containing 0.05% Tween-20 (Sigma) (PBST) and blocked with 10% non-fat dry milk powder (Quality Biological) in PBST overnight at 4°C. Sera was diluted in 5-fold increments starting with a 1:50 dilution in PBST, added to the LPS-coated plates, and incubated at 37°C for 2 hours. Plates were washed with PBST. Incubation for 1 hour with secondary HRP-conjugated antibodies, goat anti-mouse IgG, IgG1, IgG2a (Southern Biotech) or goat anti-mouse IgA (Invitrogen) was followed by a 15 minute room temperature incubation with 3,3ʹ,5,5ʹ-tetramethylbenzidine (TMB) substrate (BD biosciences) prepared according to manufacturer’s protocol. KPL TMB stop solution (Sera Care) containing 1% HCl was added to each well, and the absorbance read at 450 nm. The endpoint titer was determined as the absorbance reading that was equal to the reciprocal dilution required for the signal to match the average blank (PBST alone, no sera). Samples were run in duplicate. Sera from days 28, 42, and 56 of the vaccine study required dilutions of 1:1250 for IgG and IgG1 titers and 1:250 for IgG2a to reach specific endpoints.

### Multiplex cytokine analysis

MSD (Meso Scale Development) V-PLEX human proinflammatory panel 1 (10-plex) was used for the analysis of the human orthologs of IFN*γ*, IL-1β, IL-2, IL-4, IL-6, CXCL8, IL-10, IL-12p70, IL-13 and TNFα from 25 μL of 6-fold dilutions of the supernatant from the stimulation of human PBMCs. Samples and calibrators were incubated at room temperature, shaking (500 RPM), for 2 hours. Plates were washed with PBST and MSD detection antibody solution, prepared according to the manufacturer’s protocol, was added and incubated shaking (500 RPM) at room temperature for 2 hours. Plates were washed with 150 μL/well PBST followed by the addition of 150 μL/well of read buffer T. Plates were immediately read on an MSD SQ 120/120MM instrument. Cytokine concentration was determined by interpolation from a standard curve generated using the provided calibrators.

## Data Availability

The datasets generated during and analyzed during the current study are available from the corresponding author on reasonable request.
